# Advances in molecular innate immune regulation in the development and therapeutic strategies of allergic rhinitis vaccines

**DOI:** 10.3389/fimmu.2026.1769878

**Published:** 2026-06-05

**Authors:** Yiting Liu, Cuida Meng, Jichao Sha, Dongdong Zhu, Qingjia Sun, Nan Wu

**Affiliations:** 1 Department of Otolaryngology Head and Neck Surgery, China-Japan Union Hospital of Jilin University, Changchun, China; 2 Jilin Provincial Key Laboratory of Precise Diagnosis and Treatment of Upper Airway Allergic Diseases, China-Japan Union Hospital of Jilin University, Changchun, China; 3 Otolaryngology Head and Neck Surgery Research Center, China-Japan Union Hospital of Jilin University, Changchun, China; 4 Phase I Clinical Trial Research Laboratory, China-Japan Union Hospital of Jilin University, Changchun, China

**Keywords:** adjuvants, allergic rhinitis, immune regulation, immunotherapy, molecular innate immunity, toll-like receptors, vaccine development

## Abstract

Allergic rhinitis (AR) is an IgE-dependent, Th2 cell-mediated disorder that significantly impairs patients’ quality of life. In recent years, molecular innate immune regulation has emerged as a pivotal area of research, especially focusing on the use of Toll-like receptor (TLR) agonists as adjuvants in allergen immunotherapy (AIT). TLRs, key components of the innate immune system, recognize pathogen-associated molecular patterns and activate dendritic cells, thereby modulating adaptive immune responses to promote anti-allergic immune deviation. This review provides a comprehensive overview of the role of TLRs in the development of AR vaccines, summarizing the progress made with various TLR agonists and their potential applications in AIT. Emphasis is placed on the immunoregulatory characteristics of these molecules and the current status of clinical trials, aiming to offer a theoretical foundation and research direction for the design of more effective and fast-acting therapeutic strategies for allergic rhinitis.

## 1 Introduction

Allergic rhinitis (AR) is a pervasive chronic inflammatory disorder of the nasal mucosa. It afflicts a substantial proportion of the global population and has shown an increasing incidence over recent decades (1, 2). AR is characterized by symptoms such as nasal congestion, sneezing, itching, and rhinorrhea, which significantly impair quality of life, disrupt sleep, and reduce work and academic productivity (3). Although conventional treatments for AR, including oral and intranasal antihistamines, intranasal corticosteroids, decongestants, and leukotriene receptor antagonists, can provide symptomatic relief, they do not modify the underlying immunopathology or prevent disease progression (4, 5).

Allergen immunotherapy (AIT) works by inducing immune tolerance through repeated exposure to specific allergens, resulting in immunological changes such as suppression of IgE-mediated mast cell and basophil activation, immune deviation from Th2 to Th1 responses, and induction of regulatory T and B cells (6–8). However, AIT’s therapeutic benefits typically manifest slowly, requiring prolonged treatment courses of three or more years, and some patients exhibit suboptimal responses or poor adherence (9, 10). The innate immune system, particularly the molecular innate immune receptors known as toll-like receptor (TLR), has attracted considerable attention for its role in orchestrating immune responses and potential as a therapeutic target (11). TLRs are pattern recognition receptors (PRRs) that detect pathogen-associated molecular patterns (PAMPs) and damage-associated molecular patterns (DAMPs), thereby bridging innate and adaptive immunity (12–14). Activation of TLRs on antigen-presenting cells (APCs) modulates downstream signaling pathways that influence T cell polarization and antibody production, processes integral to allergic inflammation and immune tolerance (15). Notably, TLR agonists have been investigated as vaccine adjuvants to enhance immunogenicity by skewing immune responses towards Th1 or regulatory phenotypes, which could counterbalance the Th2 bias characteristic of allergic diseases (16).

For this narrative review, we searched PubMed, Web of Science, and Embase using the keywords “allergic rhinitis”, “Toll-like receptors”, “TLR agonists”, “allergen immunotherapy”, “adjuvants”, and “vaccine”. We included full-text English articles published between 2000 and 2026, focusing on TLR signaling, innate immune regulation, clinical trials of TLR agonist-adjuvanted AIT, and nanodelivery systems for AR. Reviews, meta-analyses, clinical trials, and fundamental mechanistic studies were prioritized to ensure comprehensive and up-to-date coverage.

This review aims to systematically summarize the current progress in molecular innate immune regulation with a primary focus on TLRs in the development of vaccines and therapeutic strategies for allergic rhinitis. By integrating basic immunological mechanisms, preclinical evidence, and recent clinical advances, we highlight the potential of TLR-targeted interventions to overcome limitations of current therapies and support the development of more effective, personalized approaches for AR management.

## 2 The functions and mechanisms of TLRs in innate immunity

TLRs are a family of transmembrane proteins that serve as critical pattern recognition receptors (PRRs) in the innate immune system, functioning as the first line of defense against invading pathogens. These receptors are broadly distributed on the plasma membrane and within endosomal compartments, enabling them to detect a wide array of microbial components (17, 18). Cell surface TLRs primarily recognize bacterial and fungal cell wall components, and flagellin, whereas endosomal TLRs, detect nucleic acids derived from viruses and bacteria internalized through endocytosis. This spatial distribution allows TLRs to survey both extracellular and intracellular pathogen-derived molecules effectively (19). Different TLR subtypes recognize distinct PAMPs, initiating specific signaling pathways that culminate in the activation of transcription factors such as nuclear factor kappa-light-chain-enhancer of activated B cells (NF-κB) and interferon regulatory factors (IRFs), leading to the production of pro-inflammatory cytokines and type I interferons (20). The ligand specificity and subcellular localization of these TLRs are tightly regulated by accessory proteins such as UNC93B1, which facilitates the trafficking of endosomal TLRs from the endoplasmic reticulum to their functional compartments (21, 22). The expression of TLRs varies among cell types and tissues, reflecting their specialized roles in immune surveillance. Notably, TLR expression can be modulated by environmental stimuli, infections, and inflammatory conditions, which influence immune responsiveness (23, 24). Recent research also highlights the functional diversity and evolutionary conservation of TLRs across species. The differential expression and localization of TLR subtypes enable the innate immune system to detect a broad spectrum of pathogens effectively, making TLRs pivotal in host defense and promising targets for therapeutic interventions in infectious, inflammatory, and neoplastic diseases (25–27).

TLRs are pivotal pattern recognition receptors that initiate innate immune responses upon recognition of pathogen-associated molecular patterns (PAMPs) (28). Upon ligand binding, TLRs activate intracellular signaling cascades primarily through two distinct adaptor protein-dependent pathways: the MyD88-dependent pathway and the TRIF-dependent pathway (29, 30). The MyD88-dependent pathway is utilized by all TLRs except TLR3, which signals exclusively via TRIF, whereas TLR4 can signal through both MyD88 and TRIF pathways. Activation of these pathways culminates in the induction of key transcription factors, notably NF-κB and interferon regulatory factors (IRFs), especially IRF3 and IRF7 (31). NF-κB activation leads to the transcription of pro-inflammatory cytokines such as tumor necrosis factor-alpha (TNF-α), interleukin (IL)-1β, and IL-6, whereas IRF activation promotes the production of type I interferons (IFNs), essential for antiviral defense (32). This signaling cascade not only initiates an immediate innate immune response but also promotes the maturation of dendritic cells (DCs), characterized by upregulation of major histocompatibility complex (MHC) molecules and co-stimulatory markers such as CD80 and CD86 (33). Mature DCs exhibit enhanced antigen-presenting capacity, thereby facilitating the activation and differentiation of naïve CD4+ T cells into various helper T cell subsets. Moreover, TLR engagement influences the balance between Th1 and Th2 responses, which is critical in allergic diseases such as AR (34). Activation of TLR4 has been shown to skew immune responses towards Th1 dominance, thereby counteracting Th2-mediated allergic inflammation.Recent studies have uncovered novel regulatory mechanisms of TLR signaling in allergic inflammation (35, 36). TLR4 gene polymorphisms are associated with elevated IL-8 and TNF levels in severe asthma, highlighting genetic modulation of TLR-mediated inflammation. Anakinra, an IL-1 receptor antagonist, inhibits canonical NF-κB activation in immune cells via mitochondrial antioxidant activity, indirectly regulating TLR downstream signaling. These findings integrate genetics, intracellular redox balance, and adaptor protein interactions, expanding the classical TLR signaling framework.

TLRs serve as pivotal molecular bridges linking innate and adaptive immunity by recognizing pathogen-associated molecular patterns (PAMPs) and initiating signaling cascades that shape subsequent immune responses (37). One critical mechanism by which TLR activation influences adaptive immunity is through the promotion of Th1 cell polarization, which counterbalances and suppresses Th2-mediated allergic reactions (38). Activation of TLRs, particularly TLR4 and TLR2 expressed on dendritic cells (DCs) and other APCs, leads to the production of pro-inflammatory cytokines such as IL-12 and type I interferons that favor Th1 differentiation (39). This Th1 skewing suppresses the Th2 cytokine milieu (e.g., IL-4, IL-5, IL-13) that underlies allergic inflammation, thereby attenuating hypersensitivity responses characteristic of AR (40). Experimental evidence shows that TLR agonists, when used as vaccine adjuvants, enhance Th1-biased immune responses and reduce Th2-driven pathology, highlighting their therapeutic potential in allergy management (41, 42).

Moreover, TLR signaling extends beyond influencing T helper cell subsets by inducing regulatory immune populations that promote immune tolerance. TLR activation can stimulate the expansion and functional enhancement of regulatory T cells (Tregs) and regulatory B cells (Bregs), both of which play critical roles in maintaining immune homeostasis and preventing excessive allergic inflammation (43, 44).

## 3 The role of TLRs in the progression of AR

**Figure 1:**
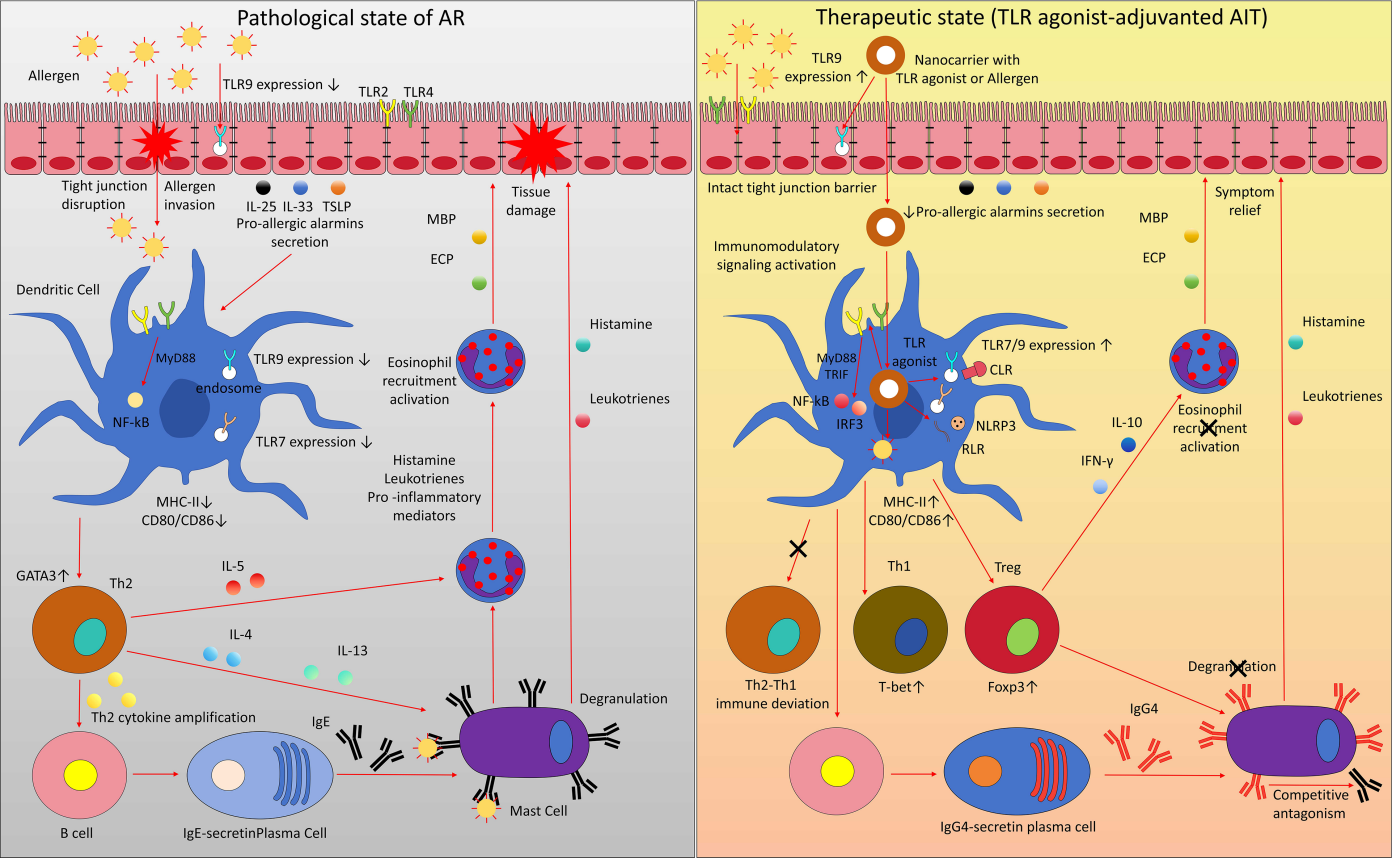
Mechanistic schematic of TLR agonist-adjuvanted AIT for AR: from pathogenic Th2 inflammation to immune tolerance.

TLRs are pivotal pattern recognition receptors that mediate innate immune responses and bridge innate and adaptive immunity. In allergic rhinitis (AR), dysregulated TLR signaling directly contributes to core clinical manifestations including nasal barrier dysfunction, eosinophilic inflammation, Th2 polarization, and persistent mucosal inflammation, all of which underlie symptom severity and disease chronicity (45). In AR, aberrant expression and dysregulated function of TLRs in the nasal mucosa have been increasingly recognized as critical contributors to disease pathogenesis.Novel clinical evidence reveals cell-type-specific TLR dysregulation in AR pathogenesis.Human nasal epithelial cells (HNECs) from AR patients exhibit reduced TLR9 expression, compromised epithelial barrier integrity, and elevated baseline secretion of IL-18 and IL-33, creating a Th2-favorable microenvironment (46). The SP-TLR4 axis is impaired, with SP inducing delayed and prolonged TLR4 upregulation in AR nasal mucosa, accompanied by decreased NEP and increased NK1R expression (47). Additionally, AR patients show 46% higher TLR9+CCR3+ granulocytes, and allergens like DAE and PPAE further upregulate basophil TLR9 expression, promoting IL-6 and IL-13 release. These findings clarify TLRs’ cell-specific roles, bridging basic research and clinical practice (48). HNECs from AR donors show differential expression of TLRs 1–6 and 9, with notably reduced TLR9 expression compared to non-atopic controls (46). This altered TLR expression is accompanied by a heightened secretion of pro-inflammatory cytokines and chemokines such as IL-1β, IL-18, IL-33, CCL-5, and IL-8 both at baseline and upon stimulation with TLR ligands or allergens (49, 50). These cytokines contribute to the establishment of a Th2-favorable microenvironment, promoting eosinophilic inflammation characteristic of AR (51). Furthermore, the nasal mucosa of AR patients show increased TLR2 and TLR4 expression, which correlates with enhanced activation of downstream signaling pathways including the NLRP3 inflammasome, exacerbating allergic inflammation. The functional consequences of TLR activation in AR are complex (52, 53). TLR engagement can modulate nasal mucosal immune responses to suppress allergic inflammation. Dysregulation of TLR signaling may contribute to impaired innate immune responses (54). In AR patients, the substance P (SP)-mediated upregulation of TLR4 in nasal epithelial cells is delayed and prolonged, contrasting with transient TLR4 induction in healthy individuals. This aberrant SP-TLR axis results in defective IL-8 production upon lipopolysaccharide stimulation, potentially contributing to prolonged infections and chronic inflammation in AR (47, 55). In addition to epithelial cells, basophils from AR patients display increased expression of TLR7 and TLR9, which are further upregulated upon allergen exposure (56). This indicates that basophils may contribute to AR pathogenesis through TLR-mediated cytokine production, including IL-6 and IL-13, thereby amplifying Th2 responses. Dendritic cells (DCs) in AR also demonstrate elevated expression of receptors such as IL-17RB, ST2, and TSLPR, which are modulated by TLR ligands and enhance their Th2-polarizing capacity, correlating with disease severity (57, 58). TLR agonists induce immune tolerance through three interconnected pathways: (1) Th2→Th1 immune deviation (59, 60): TLR4/TLR9 activation in DCs upregulates IL-12 and IFN-γ, which directly inhibit GATA3 (Th2 transcription factor) and promote T-bet (Th1 transcription factor); (2) Treg/Breg induction: TLR signaling enhances Foxp3 expression in Tregs via NF-κB-dependent IL-10 secretion, and induces Bregs to produce IL-35, suppressing effector T cell activity (61–63); (3) Protective antibody isotype switching (64, 65): TLR9 agonists promote B cell differentiation into IgG4-secreting plasma cells, and IgG4 competes with IgE for allergen binding, blocking mast cell degranulation.The three core pathways of immune tolerance induction by TLR agonists and the dual regulatory role of TLRs in AR pathogenesis are illustrated in (**Figure 1**). Specifically, the left panel visualizes the untreated AR state characterized by unimpeded Th2 polarization and IgE-mediated inflammation, while the right panel contrasts the TLR-treated state with restored immune balance, making the therapeutic shift more intuitive.

TLRs play a pivotal role in orchestrating immune deviation that is critical for alleviating allergic inflammation, especially in AR (66). TLRs, as key innate immune sensors, recognize conserved microbial components and initiate signaling cascades that shape downstream adaptive immunity (67). In the context of allergy, TLR activation promotes a shift from the pathogenic Th2-dominated immune profile—characterized by IgE production and eosinophilic inflammation—toward a Th1-skewed and regulatory T cell (Treg)-mediated immune response, which is essential for immune tolerance and symptom relief (68). Experimental evidence highlights that TLR agonists can induce the production of cytokines such as IFN-γ and IL-12, which antagonize Th2 cytokines like IL-4, IL-5, and IL-13, thereby suppressing the allergic cascade (69). Moreover, TLR stimulation enhances the induction and function of Tregs, which secrete immunosuppressive cytokines such as IL-10 and TGF-β, further dampening allergic inflammation and promoting long-term tolerance (70, 71). This immunomodulatory effect of TLRs is especially relevant in AIT, where TLR agonists serve as adjuvants to accelerate and potentiate the therapeutic immune deviation from Th2 to Th1 and regulatory responses, leading to sustained clinical remission. Clinical and preclinical studies have exhibited that synthetic agonists targeting TLR4 and TLR9, among others, can effectively enhance the efficacy of AIT by promoting these immune shifts (72, 73). However, the TLR-mediated immune modulation is complex and influenced by factors such as the specific TLR subtype engaged, the nature of the ligand, and the local microenvironment within the nasal mucosa (74, 75). Notably, in AR patients, dysregulation of TLR expression and signaling pathways has been observed, such as altered TLR4 responses to Substance P, which may contribute to the impaired innate immune defense and prolonged inflammation characteristic of allergic disease (76). Understanding these nuances is crucial for optimizing TLR-targeted therapies. Overall, TLR agonists represent a promising strategy to reprogram the allergic immune response by promoting Th1 and regulatory pathways, thereby suppressing Th2-driven allergy and achieving immune tolerance in AR and related diseases (77).

## 4 Research progress of TLR agonists as adjuvants in AR vaccines

TLR agonists have demonstrated superior capacity to activate key immune cell interactions that are essential for robust and balanced adaptive immunity. This synergistic activation of innate and adaptive immunity is not observed with alum adjuvants, which do not directly stimulate TLR pathways (78). Additionally, TLR agonists can be covalently linked to antigens to ensure co-delivery and synergistic immune stimulation, as demonstrated in novel vaccine constructs incorporating TLR7/8 and invariant natural killer T (iNKT) cell agonists, which elicit potent antibody responses with enhanced subclass diversity and functional activity (79, 80). This precise immunostimulation contrasts with the more generalized and less predictable immune activation by alum. In terms of safety and patient compliance, TLR agonists offer distinct advantages (81, 82). Traditional alum adjuvants are associated with local injection site reactions and, in some cases, systemic side effects that may reduce patient adherence to immunotherapy regimens (83). TLR agonists, particularly those derived or engineered for low toxicity, have demonstrated favorable safety profiles while providing enhanced immunogenicity (84). For instance, Mycobacterium tuberculosis Hsp70 (DnaK), which interacts with TLR4, has shown immunostimulatory effects with low toxicity and the ability to remodel the tumor immune microenvironment, suggesting potential for safe adjuvant use (85). Furthermore, the use of TLR agonists in advanced vaccine platforms, such as self-assembling nanovaccines incorporating TLR7/8 agonists and immune modulators, has resulted in improved antigen uptake, DC maturation, and antigen cross-presentation, leading to robust immune responses with reduced side effects. These innovations highlight the potential for TLR agonists to enhance immunotherapy efficacy while minimizing adverse events (86, 87). The distinct immunomodulatory profiles of different TLR subtypes and their agonists are summarized in **Table 1**, highlighting the subtype-specificity that guides adjuvant design for AR vaccines.

**Table 1:** Functional comparison of major TLR subtypes, agonists, signaling pathways and immunomodulatory effects in allergic rhinitis.

TLR subtype	Representative agonists	Primary target cells	Key immunomodulatory effects in AR	Evidence level & experimental model
TLR2	Bacterial lipoprotein derivatives	DCs	Induces Th1 polarization; promotes regulatory pathways to mitigate inflammation	Preclinical (in vitro human DCs, mouse model)
TLR4	MPLA, INI-2004, M. tuberculosis Hsp70	DCs, nasal epithelial cells	Strong Th1 skewing (↑IFN-γ/IL-12); suppresses Th2 cytokine production	Preclinical + Clinical trial evidence
TLR5	Flagellin derivatives	DCs	Shifts immunity toward Th1 dominance; enhances CD8^+^ T cell priming	Preclinical (mouse model, in vitro DCs)
TLR7	Imiquimod, GSK2245035	Plasmacytoid DCs, basophils	Stimulates type I IFN production; promotes Th1-biased immunity	Preclinical + clinical data
TLR9	CpG oligodeoxynucleotides (ODNs)	Plasmacytoid DCs, B cells	Induces Treg/Breg differentiation; drives IgE→IgG isotype switching (blocking antibodies)	Preclinical + clinical trial evidence

TLR4 recognizes lipopolysaccharide (LPS) components derived from Gram-negative bacteria, and its agonists, including LPS derivatives like monophosphoryl lipid A (MPLA), are known to promote robust Th1 immune responses (88). This Th1 skewing is characterized by the induction of pro-inflammatory cytokines such as IFN-γ, IL-12, and TNF-α, which counterbalance the Th2-dominated allergic inflammation typical of AR (89). MPLA, a detoxified LPS derivative, has been formulated in particulate adjuvants such as saponin/MPLA nanoparticles (SMNP), which enhance antigen delivery to draining lymph nodes and potentiate germinal center B cell and T follicular helper cell responses, thereby promoting durable adaptive immunity (90). Furthermore, synthetic TLR4 agonists like INI-2004 have shown efficacy in reducing allergic airway inflammation and Th2 cytokine production in murine models of allergic sensitization, underscoring their therapeutic potential in AR (91). pG oligodeoxynucleotides, synthetic ligands mimicking bacterial DNA, activate TLR9 predominantly expressed in plasmacytoid dendritic cells and B cells (92). TLR9 agonists induce a regulatory immune milieu by promoting the differentiation of regulatory T cells and facilitating antibody isotype switching from IgE to IgG subclasses, particularly IgG2a in mice, which function as blocking antibodies in allergy. In murine models, CpG-based adjuvants have enhanced Th1 responses and suppressed IgE-mediated allergic inflammation (93, 94). Moreover, co-delivery of TLR9 agonists with antigens using nanocarriers, such as alginate nanovaccines, has facilitated dendritic cell maturation and cytotoxic T lymphocyte induction, highlighting their utility in immunomodulation (95). However, some studies report that TLR9 agonists may not significantly boost humoral responses when combined with live vector vaccines, indicating the need for optimized adjuvant combinations (96). Agonists targeting TLR2, TLR5, and TLR7 have demonstrated promising anti-allergic effects *in vitro* and in animal models. TLR2 agonists, often bacterial lipoprotein derivatives, can induce Th1 responses and regulatory pathways that mitigate allergic inflammation (72). TLR5 agonists, exemplified by flagellin derivatives, have been shown to modulate the tumor microenvironment and enhance CD8+ T cell priming, suggesting their capacity to shift immune responses towards Th1 dominance and cytotoxicity. TLR7 agonists, such as imiquimod and synthetic small molecules like GSK2245035, stimulate type I interferon production and promote Th1-biased immunity (97, 98). Additionally, TLR7 expression is upregulated in basophils of AR patients, and allergen exposure further enhances TLR7 expression and cytokine production, implicating TLR7 in allergic pathophysiology (99). TLR agonists, particularly those targeting TLR4 and TLR9, have advanced into clinical trial phases and showed promising safety profiles alongside preliminary efficacy in various immunotherapeutic contexts. In the context of allergic diseases, TLR agonists administered as adjuvants have demonstrated the capacity to modulate immune responses away from pathogenic Th2 profiles toward protective Th1 and regulatory phenotypes (100, 101). These findings underscore the utility of TLR agonists in not only enhancing the magnitude but also the quality of immune responses elicited by immunotherapies.

Other PRRs have emerged as promising adjuvants for AR immunotherapy, addressing key limitations of TLR agonists. NLRP3 agonists activate the inflammasome to drive IL-1β and IL-18 secretion, which promotes Th1/Treg polarization without eliciting systemic cytokine storms (102, 103). C-type lectin receptor (CLR) agonists target DC-SIGN on DCs, enhancing antigen cross-presentation and mucosal IgA production (104). RIG-I-like receptor (RLR) agonists also stimulate type I interferon secretion, which synergizes with TLR4 agonists to augment the efficacy of allergen AIT (105). Collectively, these non-TLR PRR agonists expand the adjuvant repertoire for AR, providing valuable therapeutic alternatives for patients unresponsive to TLR-based immunotherapies.

### 4.1 Critical comparison of TLR agonists in AR immunotherapy

TLR-based adjuvants exhibit distinct advantages over conventional alum adjuvants in terms of clinical safety and efficacy, supported by robust evidence from recent clinical trials in AR. For TLR4 agonists, compelling clinical data have been generated across multiple investigations. A phase I randomized, double-blind, placebo-controlled trial of the TLR4 agonist CRX-675 in patients with ragweed-induced seasonal AR reported no serious or severe adverse events (106). Among the 64 treated patients, 77% of adverse events were mild, with a safety profile comparable to placebo and no evidence of dose-related toxicity. Notably, the 100 μg dose of CRX-675 induced consistent reductions in nasal symptom scores at 1 and 14 days post-ragweed challenge, confirming its potential therapeutic activity in AR. Another phase I/IIa double-blind, placebo-controlled study evaluated sublingual immunotherapy (SLIT) adjuvanted with MPL in patients sensitized to grass pollen (107). Active treatment groups exhibited local and systemic adverse event profiles comparable to placebo, with no serious adverse events reported; most treatment-related events were mild local reactions that resolved spontaneously. Furthermore, the two groups receiving SLIT with the highest MPL doses (52.5 μg) achieved negative nasal challenge test rates of 47% and 44% at week 10—markedly higher than the 20% rate in the placebo group—along with earlier elevations in grass pollen-specific IgG and blunted increases in allergen-specific IgE. An *in vitro* study of birch pollen allergoid combined with MPL further confirmed the safety of this TLR4 agonist: it enhanced HLA-DR expression on B cells from allergic patients to potentiate antigen presentation, while reducing basophil activation by 100- to >10, 000-fold relative to native birch pollen (108).

Clinical data similarly highlight the favorable clinical performance of TLR9 agonists in the management of AR. A phase IIb randomized, double-blind, placebo-controlled multicenter trial of CYT003-QbG10—a virus-like particle encapsulating A-type CpG oligonucleotide G10—in patients with HDM-induced perennial allergic rhinoconjunctivitis demonstrated excellent safety and tolerability (109). The most common adverse events were mild-to-moderate injection site reactions, with no drug-related serious adverse events reported throughout the trial. In terms of efficacy, the high-dose CYT003-QbG10 group showed a statistically significant reduction in the average combined symptom and medication score (ACS), with a markedly greater degree of improvement compared with the placebo group. Additionally, the high-dose treatment group achieved significantly greater improvements in patient quality of life and a more prominent elevation in allergen tolerance on conjunctival provocation testing (CPT), while no meaningful changes in these endpoints were observed in the placebo group. A phase I/IIa open-label study further evaluated QbG10 as an adjuvant for subcutaneous immunotherapy (SCIT) with HDM allergen extract in HDM-allergic patients (110). QbG10 was well-tolerated in this cohort, with no severe adverse events reported; all local reactions including pain, erythema and swelling were mild and transient, with their incidence declining along with continued treatment. Clinically, patients achieved substantial resolution of rhinitis and comorbid allergic asthma symptoms shortly after treatment initiation, and this clinical benefit was sustained for a long period after the completion of treatment. CPT further confirmed a marked elevation in allergen tolerance in treated patients, alongside reduced skin reactivity to HDM, favorable upregulation of allergen-specific IgG levels, and a benign dynamic change in IgE levels characterized by a transient rise followed by a sustained decline.

TLR agonists display distinct profiles in efficacy, safety and clinical applicability for AR immunotherapy, with subtype-specific characteristics guiding rational clinical selection. TLR4 agonists mediate potent Th1 polarization and yield durable long-term clinical efficacy, with only a minimal risk of mild systemic inflammatory events. TLR7 agonists enable more rapid symptom relief and sustained allergen hyporesponsiveness, yet their clinical application is limited by dose-dependent flu-like symptoms and local mucosal irritation. TLR9 agonists are characterized by the preferential induction of regulatory immunity, driving robust IgG4 production and Treg/Breg differentiation, which renders them particularly well-suited for AR patients with comorbid asthma, albeit with a relatively delayed onset of clinical action (**Table 2**). Notably, combinatorial TLR agonist strategies exert synergistic immunomodulatory effects, with therapeutic efficacy significantly outperforming monotherapies in the management of refractory AR. Ultimately, the rational selection of TLR agonists should be guided by individual patient phenotypic profiles: TLR4 agonists are more suitable for patients with mild-to-moderate AR, TLR9 agonists for those with severe AR and comorbid asthma, TLR7 agonists for scenarios requiring rapid symptomatic control, and combinatorial regimens for patients with refractory disease (110–115).

**Table 2:** Clinical trial summary of representative TLR agonists for allergic rhinitis.

TLR agonist	Target TLR	Administration route	Study population	Main efficacy outcomes
CRX-675	TLR4	Intranasal	Ragweed-induced seasonal AR patients	↓ Nasal symptom scores at Day 1 & 14 post-challenge; no dose-limiting toxicity
MPLA-adjuvanted SLIT	TLR4	Sublingual	Grass pollen-allergic AR patients	↑ Negative nasal challenge rate; ↑ specific IgG, ↓ IgE rise
CYT003-QbG10	TLR9	Subcutaneous	HDM-induced perennial allergic rhinoconjunctivitis patients	↓ ACS, ↑ QoL and allergen tolerance vs. placebo; no changes in placebo group
QbG10-adjuvanted SCIT	TLR9	Subcutaneous	HDM-allergic patients with/without comorbid asthma	Rapid rhinitis symptom relief; sustained asthma improvement; ↓ skin reactivity; ↑ IgG with transient then ↓ IgE

### 4.2 Nanotechnology-enhanced delivery of TLR agonists

The application of nanotechnology in the delivery of TLR agonists represents a transformative approach to vaccine development, particularly by addressing critical challenges such as poor stability, rapid systemic clearance, and off-target inflammatory effects associated with free-form TLR agonists (116). Nanocarriers provide a versatile platform to encapsulate or conjugate TLR agonists, thereby protecting them from enzymatic degradation and enabling controlled release at specific immune sites, such as lymph nodes or APCs (117). Nanoparticle platforms also enable co-delivery strategies that combine antigens with TLR agonists to synergistically enhance immune responses. This co-delivery approach leverages the natural lymphatic drainage and cellular uptake mechanisms to concentrate immune stimulation where it is most effective, thereby enhancing vaccine potency and safety (79, 118).

Nanocarriers effectively overcome key challenges associated with agonist delivery in AR immunotherapy, namely poor stability, rapid systemic clearance and off-target inflammatory effects. Their transformative value for this application stems from three mechanistically distinct advancements: targeted delivery, controlled release and synergistic co-delivery. Targeted nanocarriers are functionalized with mucosal cell-specific ligands to facilitate their selective accumulation in nasal epithelial cells and antigen-presenting cells, thus minimizing systemic exposure while elevating local agonist concentrations (119). Controlled release systems capitalize on physiological microenvironmental cues (e.g., endosomal pH gradients) to mediate stimuli-responsive agonist release, which prolongs receptor engagement and amplifies immunomodulatory activity without the toxic effects associated with burst release (120). Synergistic co-delivery platforms encapsulate multiple bioactive molecules (e.g., TLR agonists and non-TLR innate immune modulators) to orchestrate complementary immune signaling pathways, thereby reinforcing Th1/Treg polarization and the induction of immune tolerance (**Table 3**) (121). Despite these notable mechanistic advantages, scalable manufacturing and consistent batch production remain key practical barriers to clinical translation. Lipid-based nanocarrier systems currently hold the greatest translational potential in this regard, supported by well-established, GMP-compliant manufacturing protocols (**Table 3**).

**Table 3:** Summary of nanocarrier delivery systems for TLR agonist-adjuvanted AR immunotherapy.

Nanocarrier type	Loaded payload	Targeting strategy	Release mechanism	Main research outcomes
Saponin/MPLA nanoparticles (SMNP)	MPLA (TLR4 agonist) + model allergen	Lymph node targeting via particulate uptake and drainage	Sustained release within draining lymph nodes	↑ Germinal center formation, Tfh responses, and allergen-specific IgG; balanced Th1/Th2 immunity
Alginate nanovaccine	CpG ODN (TLR9 agonist) + allergen	Mucosal retention via mucoadhesive properties	pH-responsive release in endosomal compartments	↑ DC maturation and antigen cross-presentation; ↑ T cell responses; ↓ allergic airway inflammation
Self-assembling peptide nanocarrier	TLR7/8 agonist + invariant NKT cell agonist + allergen	Cell-specific uptake by APCs via receptor-mediated endocytosis	Stimuli-responsive release triggered by endosomal pH	Potent, diverse antibody responses; ↑ mucosal immunity; ↓ systemic cytokine release risk
Liposomal delivery system	TLR4 agonist (Hsp70) + allergen	Nasal epithelial cell targeting via surface ligand functionalization	Controlled release via lipid bilayer degradation	↑ Antigen uptake by nasal epithelial cells/DCs; local Th1 polarization; improved safety

## 5 Future challenges and research directions

The ongoing development and screening of novel TLR agonists represent a critical frontier in immunotherapy, particularly for diseases such as AR where modulation of innate immunity can reshape adaptive responses. Recent advances illustrate innovative strategies to improve TLR agonist performance, including molecular modifications and engineered delivery systems. This approach highlights the potential of combining TLR agonists with immune checkpoint targeting to enhance therapeutic efficacy while minimizing systemic toxicity (86, 122). Similarly, the optimization of nanocomplex particulate adjuvants that target mast cells and TLR9 has yielded broadly active intranasal influenza vaccines with improved mucosal immunity and safety profiles, achieved through screening and engineering of peptide agonists and CpG variants. At the molecular level, chemical strategies will be employed to modulate TLR agonist activity, including prodrug and antedrug designs that respond to biological stimuli, and conjugation with other immunotherapeutic moieties to enhance targeting and efficacy (86, 123). These innovations collectively address challenges such as cytokine storms and chronic inflammation associated with nonspecific TLR stimulation, thereby improving the therapeutic index.

Notably, nanocarriers resolve the cytokine storm paradox through two core mechanisms: first, mucosal cell-specific ligand-mediated targeted delivery enriches agonists at nasal epithelial cells and APCs, limiting systemic spread; second, stimuli-responsive controlled release enables localized TLR activation. This avoids excessive pro-inflammatory cytokine secretion that triggers systemic off-target inflammation while retaining sufficient potency to drive Th2-to-Th1 immune deviation.

From a regulatory and manufacturing standpoint, self-assembling nanovaccines present unique challenges compared to conventional alum-based vaccines. Their intricate nanostructures demand stricter quality control to ensure batch consistency, and current good manufacturing practice protocols for lipid/or peptide-based nanocarriers are less mature than those for alum adjuvants. Furthermore, regulatory authorities lack standardized safety assessment frameworks for nanoscale adjuvants—particularly regarding long-term biocompatibility—potentially delaying their clinical translation.

## 6 Discussion

In conclusion, the evolving understanding of molecular innate immunity, particularly the pivotal role of TLRs in the immunoregulation of AR, marks a significant advancement in the field of allergy and immunotherapy. The intricate interplay between TLR-mediated signaling pathways and allergic inflammation provides a robust framework for developing novel adjuvant strategies aimed at enhancing vaccine efficacy. The dual capacity of TLR agonists to modulate immune responses—shifting the balance from a pathogenic Th2-dominated profile towards immune tolerance—underscores their therapeutic potential in AR management (124). This immunomodulatory effect not only promises to shorten treatment duration but also to improve clinical outcomes, addressing longstanding challenges in allergen-specific immunotherapy.

Beyond core considerations of therapeutic efficacy and safety, the rational selection of TLR agonists for AR immunotherapy must be guided by patient phenotypic stratification. TLR4 agonists are well-suited for managing mild-to-moderate AR in patients without comorbidities, given their potent Th1-polarizing activity that elicits rapid symptomatic relief with minimal systemic immune-related risks. In contrast, TLR9 agonists represent a more appropriate therapeutic choice for patients with severe AR and concomitant asthma: their ability to robustly induce regulatory T and B cell subsets and drive protective IgG4 isotype switching exerts a dual inhibitory effect on nasal mucosal inflammation and airway hyperresponsiveness, albeit with a relatively delayed clinical onset that imposes greater demands on patient treatment adherence. For patients with refractory AR unresponsive to single-agent TLR agonist therapy, combinatorial immunomodulatory strategies integrating distinct TLR-mediated signaling pathways harness synergistic effects to achieve more profound and sustained immune deviation, thereby overcoming the inherent limitations of monotherapeutic approaches.

Taken together, rational selection and clinical application of TLR agonist-adjuvanted vaccines, guided by phenotypic stratification of patients, offers a paradigm-shifting strategy to overcome the core limitations of current allergic rhinitis (AR) management. By leveraging the intrinsic immunoregulatory capacity of the innate immune molecular network, this approach directly targets the root immunopathology driving AR, while advancing the field toward the ultimate clinical goal: transitioning this chronic, debilitating condition into a controllable, and even preventable, disorder.
